# Chemo-radiotherapy after neoadjuvant chemotherapy and radical hysterectomy in women with stage IB-IIB cervical cancer: Do we need to change the therapeutic approach? A cohort study

**DOI:** 10.1016/j.sipas.2025.100284

**Published:** 2025-04-28

**Authors:** Somayeh Nikfar, Azam Sadat Mousavi, Setareh Akhavan, Shahrzad Sheikhhasani, Amir Almasi-Hashiani, Ramin Parvizrad, Narges Zamani

**Affiliations:** a Department of Obstetrics and Gynecology, Taleghani Hospital, Arak University of Medical Sciences, Arak, Iran; b Department of Oncologic Gynecology, Vali-Asr Hospital, Imam Khomeini Hospital Complex (IKHC) Tehran University of Medical Sciences, Tehran, Iran; c Department of Epidemiology, Arak University of Medical Sciences, Arak, Iran; d Department of Emergency Medicine, Arak University of Medical Sciences, Vali-Asr Hospital, Arak, Iran

**Keywords:** Chemo-radiotherapy, Neoadjuvant chemotherapy, Radical hysterectomy, Cervical cancer, Progression-free survival

## Abstract

**Background:**

Chemoradiotherapy is recommended as the standard treatment for advanced cervical cancer, and neoadjuvant chemotherapy (NACT) can be beneficial for patients on long radiotherapy waiting lists. This study aimed to evaluate the need for chemoradiotherapy after NACT and radical hysterectomy in women with stage IB-IIB cervical cancer.

**Methods:**

This was a retrospective, cohort study. All patients in the gynecologic oncology clinic of Imam Khomeini Hospital, Tehran, Iran, who were diagnosed with stage IB-IIB cervical cancer and treated with NACT and radical hysterectomy between 2010 and 2020, were included in this study. The records of all the patients who met the inclusion criteria were evaluated during the study period. Outcomes of interest and progression-free survival (PFS) were also assessed.

**Results:**

In this study, the clinical files of 613 patients with cervical cancer were studied, and among them, 63 patients (10.2%) underwent NACT. Eighteen patients (33.3%) did not require another treatment modality after chemotherapy and radical hysterectomy, while 66.7% (36 cases) of patients required chemoradiotherapy after NACT and radical hysterectomy, and recurrence was observed in 11.6% (5 cases) of patients. The 1-, 5- and 10-year PFS rate was 97.6% (95% CI: 84.2–99.6), 89.5% (95% CI: 74.4–95.9) and 89.5% (95% CI: 74.4–95.9), respectively.

**Conclusions:**

It can be concluded that a significant percentage of patients who are candidates for NACT followed by radical hysterectomy would require another modality of treatment, which is chemoradiotherapy; therefore, it is recommended that by conducting prospective studies, in addition to investigating this issue, the choice of the first method of patient treatment in these stages should be reconsidered so that patients do not suffer from two treatments and related complications, and undergo chemoradiotherapy from the beginning.

**Abbreviations**
NACT: Neoadjuvant chemotherapy
S: Surgery
CCRT: Adjuvant concurrent Chemoradiation therapy,
PFS: Progression-free survival,
CI: Confidence interval

## Introduction

Cervical cancer is the second leading cause of cancer in less-developed countries and the third leading cause of cancer in women worldwide. Unfortunately, most patients with cervical cancer in developing countries are referred to the advanced stages [1,2]. Cervical cancer can be treated with surgery or radiotherapy with or without chemotherapy, depending on the size of the lesion, stage of the disease, histological features, lymph node involvement, risk factors for surgery or radiotherapy, and the patient's preferred treatment [3]. Chemoradiotherapy is recommended as the standard treatment for advanced cervical cancer, but radiotherapy facilities are not available in developing countries, and neoadjuvant chemotherapy can be beneficial for patients on long radiotherapy waiting lists [4,5].

Currently, there is no conclusive evidence for the use of this therapeutic approach, and further studies are needed. Numerous studies have shown that neoadjuvant chemotherapy is effective in reducing tumor size and eliminating micro-metastases and the possibility of surgery and reducing the disease stage in surgery [6]. It is estimated that more than 38% of cervical cancers are diagnosed at stage IB2-IIB. However, the treatment strategy for stage IB2-IIB, and especially stage IIB, is controversial. Although most patients with stage IB2-IIB cervical cancer initially respond to conventional therapy, which is concomitant chemotherapy based on cisplatin and external radiotherapy followed by brachytherapy (CCRT), 22% −41% of patients still experience recurrence [[7], [8], [9], [10]].

In addition, this treatment is associated with early and long-term toxicities including radiation cystitis, radiation-induced enterocolitis, vaginal stenosis, and pelvic adhesions. Therefore, physicians are actively considering more effective treatment options. Neoadjuvant chemotherapy (NACT) followed by radical surgery (hysterectomy plus pelvic lymph node dissection) (NACT + S) is the most researched treatment method and has attracted the most attention because, in addition to disease control, it is less toxicity [11]. Many studies have shown that NACT + S can reduce tumor size, improve R0 resection, reduce the risk of intraoperative spread, reduce the incidence of postoperative complications, and even improve survival outcomes compared with surgery or radiation therapy. However, this therapeutic method also has disadvantages such as prolonged treatment, increased medical costs, and potential tumor progression due to insensitivity to chemotherapy. In addition, some studies have demonstrated that NACT + S has no survival benefit. Therefore, controversy remains regarding NACT + S treatment [4,[12], [13], [14], [15], [16]].

Treatment of cervical cancer can affect all aspects of a woman's life, including her physical and mental functioning. One of the most important activities that changes during cervical cancer and radiotherapy is the sexual function of women. The results of some studies in this field have shown that both surgical and radiotherapy methods are harmful and have different effects on body function, especially on female sexual function. Further studies have raised the question of which treatment method is better for patients with cervical cancer stage IIB-IB2 [[17], [18], [19]]. In addition, improving the quality of life of patients, in addition to increasing survival in patients with cervical cancer, is a highly important issue. Therefore, in this study, we aimed to evaluate the need for chemoradiotherapy after neoadjuvant chemotherapy and radical hysterectomy in women with stage IB-IIB cervical cancer to prevent the imposition of both treatments.

## Materials and methods

**Study design**: This was a retrospective cohort study. All patients in the gynecologic oncology clinic of Imam Khomeini Hospital who were diagnosed with stage IB-IIB cervical cancer and were treated with NACT and radical hysterectomy between 2010 and 2020 were included in this study. The records of all the patients with cervical cancer who met the inclusion criteria were evaluated during the study period.

**Ethical considerations**: All ethical codes related to the human studies were considered. Patient information was kept confidential. In addition, the collected information of patients was anonymous, and in cases where patients were contacted by phone, they were asked if they wished to participate in the study. This study was approved by the ethics committee of Tehran University of Medical Sciences (ethics code IR.TUMS.IKHC.REC.1399.382).

**Inclusion and exclusion criteria**: Inclusion criteria comprised All patients were diagnosed with primary cervical cancer stage IB-IIB, treated with neoadjuvant chemotherapy and radical hysterectomy, and available clinical records. All patients who received neoadjuvant chemotherapy (NACT) followed by surgery were administered a combination regimen of paclitaxel and cisplatin. Following this chemotherapy regimen, patients underwent radical hysterectomy. The exclusion criteria were incomplete data recorded in the file and incomplete treatment period before the start of the study.

**Study procedure**: The files of patients with cervical cancer who were referred to the oncology clinic were reviewed in the desired period. The files of eligible patients were retrospectively studied, and the required data, including patient age, parity, disease stage, type of neoadjuvant chemotherapy regimen used along with the dates of start and end, and postoperative pathology, were extracted from the files based on a questionnaire designed by the researcher. In addition, progression-free survival was evaluated. In cases where necessary, the telephone number in the patient's file was used to complete the data and people were contacted. In cases where the patients' information was incomplete, this information was considered missing data, and other information of that patient was used in other analyses.

The outcomes studied in this research included recurrence, recurrence time, recurrence treatment, chemoradiotherapy sessions, and progression-free survival, which were collected from patient records or by telephone calls. Required information was collected using a checklist designed by the researcher. Disease recurrence was evaluated based on clinical symptoms and imaging results. Progression-free survival was also evaluated based on the duration of survival after initial treatment of the disease without any signs or symptoms of the disease.

Owing to the nature of the study, all cases of cervical cancer that met the inclusion criteria were included in the study over a period of 10 years. It should be noted that this hospital is considered a referral center for cancer in Iran.

**Statistical analyzes**: Mean (standard deviation) and frequency (percentage) were used to describe the quantitative and qualitative data, respectively. To evaluate progression-free survival (PFS), the Kaplan-Meier method was used, and the survival rate was reported using a survival curve. All analyses were performed using the Stata software version 13 (Stata Corp, College Station, TX, USA).

## Results

In this study, clinical files of 613 patients with cervical cancer referred to the oncology clinic of Imam Khomeini Hospital in Tehran during 2010 to 2020 were studied and among them, 63 patients (10.2% of patients) underwent NACT and by considering the reports and inclusion criteria, 48 patients (76.1% of NACT recipients) were included in the study, all of whom underwent NACT and radical hysterectomy. Of the remaining 15 patients, the date of surgery was unknown in six patients, and due to the lack of follow-up, the need for chemoradiotherapy was unknown in these cases. In two patients, radical surgery could not be performed due to severe intra-abdominal adhesions, and these patients were considered candidates for chemoradiotherapy alone. In addition to the NACT regimen, four patients received radiotherapy and underwent radical hysterectomy, one of whom underwent brachytherapy after surgery, one received radiotherapy up to the para-aortic region, and the other two patients were not suitable candidates for radical hysterectomy after receiving NACT and as a result, were candidates for chemoradiotherapy. One of them became a candidate for total hysterectomy after completing radiotherapy due to the presence of atypical cells in the cervix.

In 63 cases that were included in the initial analysis, the mean age of patients was 45.22 years (standard deviation 9.9 years). The mean gravidity of the patients was 4.11 (with a standard deviation of 2.6). Regarding the stage of the disease at the time of diagnosis, 27 cases (44.3%) were stage Ib2, 13 cases (21.3%) were stage IIb, and 8 cases (13.1%) were stage IIa. In terms of the NACT regimen, all patients were treated with taxol and cisplatin regimens, and four patients (6.3%) underwent radiotherapy in addition to chemotherapy. In 59 patients (95.2%), three NACT courses were used. 45 cases (90%) were squamous cell carcinoma (SCC) and 5 cases (10%) were adenocarcinoma. Regarding lymphovascular space invasion, 25 cases (53.2%) were negative and 22 cases (46.8%) were positive (Table 1). The tumor invasion depth was less than 50% in 25.5% (12 patients), more than 50% in 53.2% (25 patients), and negative in 21.3% (10 patients). In terms of margin, lymph node, lower segment, and parameter involvement, 44 (93.6%), 40 (85.11%), 43 (91.5%), and 46 (97.9%) patients were negative, respectively. The pathological risk of patients was also assessed, and the results showed that this risk was high in eight patients, moderate in 20 patients, and low in 18 patients.

**Table 1:** Frequency distribution of demographic and clinical variables in patients diagnosed with stage IB-IIB cervical cancer treated with NACT and radical hysterectomy.

Variables		N (%)
Age	Mean (S.D.)	45.22 (9.7)
Gravidity	Mean (S.D.)	4.11 (2.6)
Stage	1B2	27 (44.26)
	1A2	1 (1.64)
	1B	3 (4.92)
	1B3	4 (6.56)
	2A	8 (13.11)
	2B	13 (21.31)
	2A2	4 (6.56)
	2A1	1 (1.64)
NACT	T+cis	59 (93.6)
	T+cis+rad	4 (6.4)
NACT courses	1	1 (1.61)
	2	1 (1.61)
	3	59 (95.2)
	4	1 (1.61)
Primary pathology	Adenocarcinoma	5 (10.0)
	SCC	45 (90.0)
LVSI	Negative	25 (53.2)
	Positive	22 (46.8)
Depth	<50%	12 (25.5)
	>50%	25 (53.2)
	Negative	10 (21.3)
Marg	Negative	44 (93.6)
	Positive	3 (6.4)
LN	Negative	40 (85.1)
	Positive	7 (14.9)
Lower Seg	Negative	43 (92.5)
	Positive	4 (8.5)
Parameter	Negative	46 (97.9)
	Positive	1 (2.1)
Pathology risk	High	8 (17.4)
	Moderate	20 (43.5)
	Low	18 (39.1)
Chemo-radiotherapy	No	18 (33.3)
	Yes	36 (66.7)
Recurrence	No	38 (88.4)
	Yes	5 (11.6)

Follow-up of patients showed that only 18 patients (33.3%) did not require another treatment modality after chemotherapy and radical hysterectomy, whereas 66.7% (36 cases) of patients required chemoradiotherapy after neoadjuvant chemotherapy and radical hysterectomy. Patients were also evaluated for recurrence, of which 88.4% had no recurrence, while recurrence was observed in 11.6% (five cases) of patients.

**Fig. 1:**
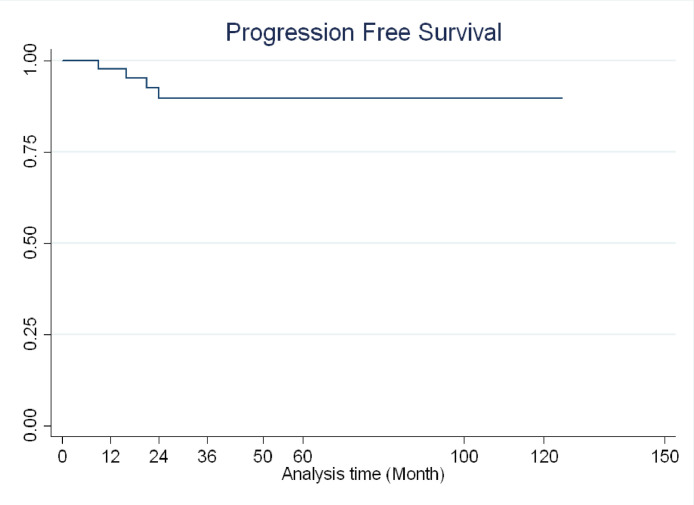
10-year progression-free survival (PFS) in patients diagnosed with stage IB-IIB cervical cancer treated with NACT and radical hysterectomy.

Fig. 1 shows the results of the Progression-Free Survival (PFS) analysis. According to this Figure, the PFS rate after one year was 97.6% (95% CI: 84.2–99.6), two-year PFS was 89.5% (95% CI: 74.4–95.9), 5-year PFS was 89.5% (95% CI: 74.4–95.9) and 10-year PFS were 89.5% (95% CI: 74.4–95.9).

## Discussion

Of the 48 patients who underwent NACT and radical hysterectomy, 29 (60.4%) required chemoradiotherapy and 19 (39.6%) did not require further treatment. The need for chemoradiotherapy in stages IIA, IB, and IIB was 33.3%, 66.67%, and 75%, respectively (*p* = 0.28). Although the differences observed in the study population were not statistically significant, this difference could be clinically significant and may indicate a possible association between disease stage and the need for chemoradiotherapy, which should be investigated in larger studies. The mean overall survival (OS) and progression-free survival (PFS) in the studied patients were 52.8 months (40.9–64.7, CI:95%) and 51.1 months (39.3–62.9, CI:95%), respectively.

The recurrence rate in the study population was 10.5% (*n* = 5). Postoperative pathology demonstrated that four of them were in the high-risk group and underwent chemoradiotherapy, and the remaining patients were in the intermediate-risk group. After surgery, she was administered radiotherapy but only received external radiotherapy and did not undergo brachytherapy, which highlights the importance of brachytherapy in preventing the recurrence of cervical cancer.

Of the cases studied, 3 died (6.2%), all of which occurred in the recurrence group. 24 months after chemoradiotherapy, recurrence with bone metastasis was observed in one patient who was referred for chemotherapy and died 24 months after recurrence (OS= 48 months); the second patient, considering the pathology after surgery, was a candidate for chemoradiotherapy who refused to receive treatment, and after 9 months, metastasis to the bladder was observed, and the patient died after 2 months (OS= 11 months); and the third patient underwent chemoradiotherapy after surgery but did not receive brachytherapy and died 8 months after completing radiotherapy with bone metastasis. The mean PFS of patients with recurrence was 17.5 months (95% CI, 7–28 months), and their OS was 25.6 months (95% CI, 3.8–46.8 months).

Nama et al. [20] conducted a non-randomized study in 2018 to evaluate the increase in morbidity due to surgery with NACT compared to primary chemoradiotherapy in patients with IB2 stage cervical cancer, and compared the results of surgery and chemoradiotherapy, and found that the data were similar for patients with stage IB2 and IIA; therefore, they concluded that to date, there is no conclusive evidence for a preferred treatment option for stage IB2 cervical cancer.

The findings of the present study showed that both treatment failure and recurrence occurred in the first two years after treatment and after two years, the PFS rate remained at 89.5% until the end of the study (end of 10 years).

In a meta-analysis published by Ye et al. [21] in 2020, it was concluded that the short-term therapeutic effects of both treatment options were similar in patients with IB2-IIB stages of cervical cancer, but the long-term effects of NACT and surgery on OS and DFS were better than those of radiotherapy alone or CCRT; However, the limitations of this meta-analysis included the relatively small number of articles reviewed (five articles), reviewing only survival rates and complications, and not reviewing the quality of life.

A meta-analysis by Cheng et al. [22] found that CCRT in patients with IB2 / IIA2 stage cervical cancer was probably the best way to improve clinical outcomes and suggested that phase III randomized trials be performed to assess this matter. In addition, Lee et al. conducted a study [23] that compared 85 patients with IB-IIB cervical cancer treated with NACT + S and 358 patients receiving CCRT, and concluded that in patients with IB-IIB cervical cancer, NACT treatment with surgery has no therapeutic advantage over CCRT.

This study has the inherent limitation of being a retrospective study, so further research with a more precise methodology is warranted. Additionally, the small sample size of the study may have reduced its statistical power, highlighting the need for further studies such as multicenter research with a larger sample size.

## Conclusion

According to the results of this study, it can be stated that a significant percentage of patients with stage IIA and IIB cervical cancer who are candidates for NACT followed by radical hysterectomy would require another modality of treatment, which is chemoradiotherapy; therefore, it is recommended that by conducting prospective studies, in addition to investigating this issue, the choice of the first method of treatment of patients in these stages should be reconsidered. By doing so, we hope to minimize the need for multiple treatments and their associated complications. Ideally, patients would undergo the most effective treatment from the onset, whether that be chemoradiotherapy or NACT followed by radical hysterectomy. This theory requires a comparative study of overall survival, PFS, and quality of life after treatment between patients receiving chemoradiotherapy and those receiving NACT and radical hysterectomy and subsequent chemoradiotherapy.

## Ethical approval

This study was conducted in accordance with the Helsinki Declaration and was approved by the Tehran University of Medical Sciences ethics committee (IR.TUMS.IKHC.REC.1399.382).

## Funding sources

Not applicable.

## Consent for publication

All the patients signed the informed consent form. A copy of the written consent is available for review by the Editor-in-Chief of this journal on request.

## Availability of data and materials

All data generated or analyzed during this study are available for review by the Editor-in-Chief of this journal on request.

## CRediT authorship contribution statement

**Somayeh Nikfar:** Writing – original draft, Data curation. **Azam Sadat Mousavi:** Supervision. **Setareh Akhavan:** Data curation. **Shahrzad Sheikhhasani:** Data curation. **Amir Almasi-Hashiani:** Methodology. **Ramin Parvizrad:** Formal analysis. **Narges Zamani:** Writing – review & editing.

## Declaration of competing interest

The authors declare that they have no known competing financial interests or personal relationships that could have appeared to influence the work reported in this paper.
